# Circular RNAs: new biomarkers of chemoresistance in cancer

**DOI:** 10.20892/j.issn.2095-3941.2020.0312

**Published:** 2021-06-15

**Authors:** Jiaqi Wang, Yi Zhang, Lianyu Liu, Ting Yang, Jun Song

**Affiliations:** 1 Department of General Surgery, The Affiliated Hospital of Xuzhou Medical University, Xuzhou 221002, China; 2 Institute of Digestive Diseases of Xuzhou Medical University, Xuzhou 221002, China

**Keywords:** Circular RNA, chemoresistance, drug resistance, cancer

## Abstract

Chemotherapeutics are validated conventional treatments for patients with advanced cancer. However, with continual application of chemotherapeutics, chemoresistance, which is often predictive of poor prognosis, has gradually become a concern in recent years. Circular RNAs (circRNAs), a class of endogenous noncoding RNAs (ncRNAs) with a closed-loop structure, have been reported to be notable targets and markers for the prognosis, diagnosis, and treatment of many diseases, particularly cancer. Although dozens of studies have shown that circRNAs play major roles in drug-resistance activity in tumors, the mechanisms by which circRNAs affect chemoresistance have yet to be explored. In this review, we describe the detailed mechanisms of circRNAs and chemotherapeutics in various cancers and summarize potential therapeutic targets for drug-resistant tumors.

## Introduction

Circular RNAs (circRNAs), which were first identified more than 4 decades ago^1^, are a class of endogenous ncRNAs that have a closed-loop structure, and lack 5′ caps and 3′ poly (A) tails^2^. Owing to their unique structure, circRNAs are more stable and less easily degraded than mRNAs^3^. Although circRNAs are abundant in eukaryotes, scientists initially considered them to have no meaningful function^4^. However, the development of high-throughput deep RNA sequencing and the application of bioinformatics technology has resulted in the confirmation of circRNAs as important molecules that are abnormally expressed and associated with poor prognosis in a variety of diseases, such as cardiovascular diseases^5^, diabetes^6^, nervous system diseases^7^, immune system diseases^8^, and cancer^9^.

Chemotherapeutics, which have been validated as conventional treatments for patients with advanced cancer, have widespread application in clinical practice. However, the development of chemoresistance, along with the need for chemotherapy treatment, is often predictive of poor prognosis^10^. Although the literature regarding drug resistance is extensive, the mechanisms involved require further clarification. In this review, we describe the relationship between circRNAs and chemoresistance, and discuss the mechanisms through which circRNAs contribute to drug resistance in different cancers.

## Biogenesis of circRNAs

**Figure 1:**
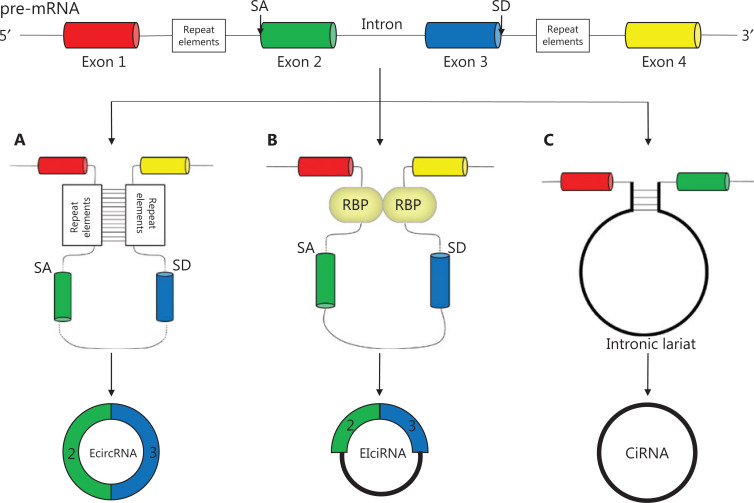
Biogenesis of circRNAs: (A, B) EcircRNAs and EIciRNAs can be generated from an SD site attacking an SA site, with sites with repeat elements close together; (C) Intronic circRNAs can be generated from intronic lariats that escape the debranching step of canonical linear splicing.

According to several studies, circRNAs are derived from canonical splice sites, and their biogenesis is dependent on the canonical splicing machinery^11,12^. However, Liang et al.^13^ have indicated that, when pre-mRNA processing events are slowed down, nascent RNA can be directed to alternative pathways that facilitate backsplicing. The main concept of backsplicing is that looping of the intron sequences flanking the downstream splice-donor (SD) site and the upstream splice-acceptor (SA) site brings these sites close together. Formation of this circular structure is mediated by base pairing between inverted repeat elements located in the upstream and downstream introns, or by the dimerization of RNA-binding proteins (RBPs) that bind particular motifs of lateral introns^14^. During backsplicing, an upstream branch point attacks a downstream SD site, which subsequently attacks an upstream SA site and results in the formation of exonic circRNAs (EcircRNAs) (**Figure 1A**) or exon–intron circRNAs (EIciRNAs) (**Figure 1B**). In addition, during exon skipping, lariat formation, in which alternative exons are spliced out of the final mRNA product and are eventually included in the excised lariat, contribute to circRNA formation when the lariat undergoes internal backsplicing^15^. Finally, intronic lariats that escape from debranching can lead to the formation of intronic circRNAs^16^ (**Figure 1C**).

## Biological functions of circRNAs in cancer

### Functions as miRNA sponges

**Figure 2:**
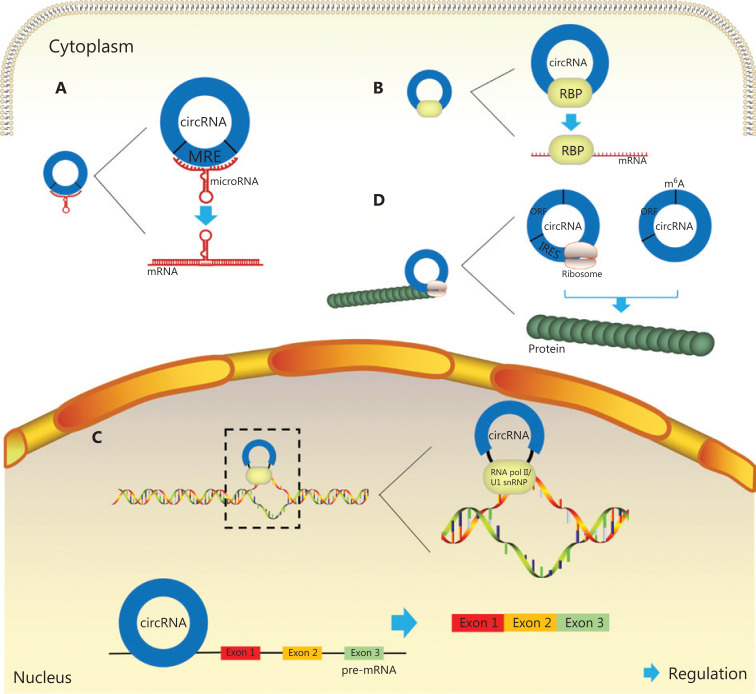
Biological functions of circRNAs in cancer: (A) Functions as miRNA sponges: miRNAs can bind miRNA response elements of circRNAs and thereby alleviate repression of downstream mRNAs; (B) Functions through RNA-binding proteins: circRNAs can bind RBPs which have binding sites affecting the expression of associated genes; (C) Functions as transcriptional regulators: circRNAs can regulate transcription by binding polymerase II or U1 snRNP complexes, as well as regulating selective splicing; (D) Functions in protein translation: circRNAs with open reading frames and internal ribosome entry sites can translate protein. Moreover, m^6^A motif, which is sufficient to initiate translation, is recognized rich in some circRNAs, so as to empower these circRNAs to translate protein.

MicroRNAs (miRNAs) are ncRNAs that negatively regulate mRNA translation through specific binding to target sites in the 3′ untranslated regions of mRNA^17^. Many reported circRNAs contain miRNA response elements, which may play roles as miRNA sponges and serve as competing endogenous RNAs, thus preventing downstream target mRNAs from undergoing miRNA repression. Generally, according to most reports, one circRNA sponges a single miRNA sequence and plays a regulatory role. For example, CDR1as, which is known as a “super-sponge,” strongly sponges miR-7, which contains more than 70 selectively conserved binding sites^18^, thereby affecting the occurrence and development of melanoma^19^, non-smallcell lung cancer^20^, and other cancers. However, some circRNAs that act as RNA sponges, such as circHIPK3 and circCCDC66, can interact and regulate multiple miRNAs simultaneously. CircHIPK3, which is derived from exon 2 of the *HIPK3* gene and consists solely of a large single exon (1,099 bp), may sponge as many as 9 miRNAs, as detected by luciferase reporter assays^21^. In addition, circCCDC66 has been reported to sponge 4 different miRNAs in various cancers^22–25^ (**Figure 2A**).

### Functions through RNA-binding proteins

Some circRNAs, similarly to their roles as miRNA sponges, bind RBPs, which play a crucial role in posttranscriptional modification and mRNA translation, and act as protein sponges affecting the expression of associated genes. A recent report has revealed that circZKSCAN1 competitively binds the RBP fragile X mental retardation protein (FMRP), thus preventing its binding to β-catenin-binding protein-cell cycle and apoptosis regulator 1 (CCAR1) mRNA and consequently leading to downregulation of the transcriptional activity of Wnt signaling targets in hepatocellular carcinoma^26^ (**Figure 2B**).

### Functions as transcriptional regulators

CircRNAs regulate transcription through 2 recognized mechanisms. In one mechanism, circRNAs, particularly EIciRNAs (which are backspliced and retain introns), bind the polymerase II complex and interact with both the U1 small nuclear ribonucleoprotein (snRNP) complex and the promoters of their encoding genes^27^. Two examples of this subgroup of circRNAs are EIciEIF3J and EIciPAIP2. If the interactions between these EIciRNAs and RNA polymerase II or U1 snRNPs are blocked, the mRNA transcription of their parental genes, eukaryotic translation initiation factor 3 subunit J (EIF3J) and poly (A)-binding protein-interacting protein 2 (PAIP2), respectively, is downregulated^28^. Furthermore, the circRNA FECR1, which is derived from backsplicing between exon 4 and exon 2 of *FLI1*, activates FLI1 expression by interacting with the *FLI1* promoter and subsequently induces extensive demethylation of CpG islands. Moreover, circFECR1 binds the promoters of *DNMT1* and *TET1*, and regulates the expression of genes associated with DNA methylation and demethylation^29^.

The other mechanism through which circRNAs (particularly EcircRNAs) regulate transcription is selective splicing. Because some circRNAs have functional binding sites, they can compete with linear mRNAs for canonical splicing and then alter the expression of their parental genes; for instance, circMbl interacts with the splicing complex, such that Mbl promotes the expression of circMbl^30^. A pioneering study on titin, which was published just before the rediscovery of circRNAs, has shown that a subset of circRNAs that originate from the I-band of the titin gene are regulated by the splicing factor RBM20^31^. These results demonstrate another aspect of the relationship between circRNAs and selective splicing (**Figure 2C**).

### Functions in protein translation

CircRNAs were previously considered to lack the ability to be translated, because they do not possess 5′ cap structures, poly (A) tails, or internal ribosome entry sites, all of which are essential for translation^32^. However, more recent studies have debunked this theory. An open reading frame in circZNF609 has been reported to be recognizable by a splicing-dependent and cap-independent mechanism^33^. The translation mechanism driven by N6-methyladenosine (m^6^A) modification has become another area of interest. YTHDC1, an m^6^A reader, has been found to recognize modifications on circNSUN2 and then facilitate circNSUN2 export from the nucleus to the cytoplasm; this activity directly increases the invasion ability of colorectal cancer (CRC)^34^ (**Figure 2D**).

## Mechanisms of chemoresistance in circRNAs

**Figure 3:**
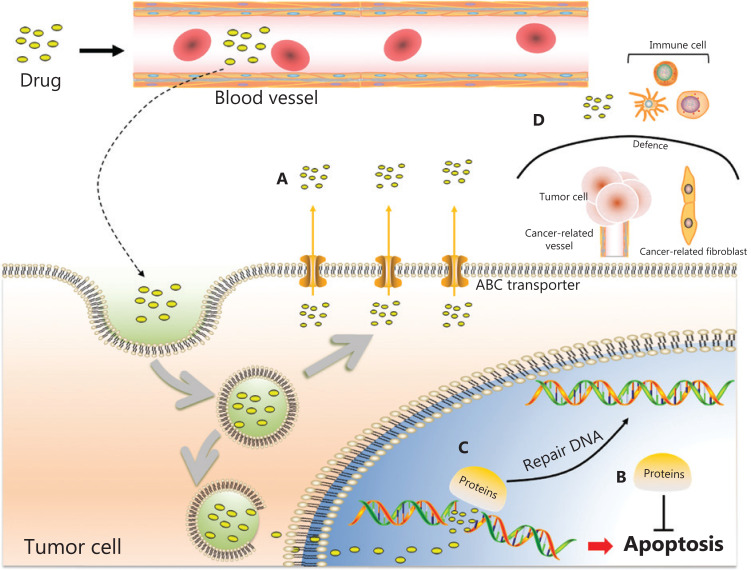
Mechanisms of chemoresistance: (A) Promoting drug excretion: ABC transporters enhance drug excretion, thus leading to low drug accumulation in cells; (B) Regulating apoptosis-related genes: upregulation of anti-apoptotic genes or downregulation of apoptotic genes allows cancer cells to proliferate indefinitely without constraint, thus resisting chemotherapy; (C) Enhancing DNA repair: some proteins contribute to repairing damaged DNA, thus resulting in the failure of drugs that destroy the structure of DNA; (D) Creating the TME: a hypoxic, acidic, inflammatory, and immunosuppressive TME with cancer-related fibroblasts and vessels can protect tumor cells from immune cells and killing by drugs.

Chemotherapy plays an indispensable role in the treatment of cancer, particularly advanced cancer. However, chemoresistance emerges and then becomes an urgent obstacle to overcome. Many reports have described the mechanisms of drug resistance in cancer, which include (1) promoting drug excretion by expressing ATP-binding cassette (ABC) transporters such as ABCB1, ABCC1, and ABCG2, which are often overexpressed in drug-resistant cancer cells and result in lower drug accumulation in cells^35^; (2) dysregulating the expression of anti-apoptotic genes (e.g., upregulating the expression of the antiapoptotic genes *Bcl-2* and *MDM2*, or repressing the expression of tumor suppressors such as *p53*), thus allowing cancer cells to proliferate indefinitely without constraint^36^; (3) enhancing the capacity for DNA repair (e.g., in CD133 positive glioma stem cells, which show increased activation of checkpoint-related proteins and other proteins involved in the DNA damage response)^37^; and (4) creating a tumor microenvironment (TME) with an altered proportion of stromal fibroblasts, vasculature, and immune cells^38^ (**Figure 3**). In addition to these mechanisms, many other factors, such as tumor heterogeneity, autophagy, and gene mutations, affect drug resistance^39–41^.

Although dozens of studies have shown that circRNAs play major roles in drug-resistance activity in tumors, the mechanisms through which circRNAs affect chemoresistance have yet to be explored. In the next section, circRNAs and their mechanisms relating to drug resistance are summarized in detail.

## The roles of circRNAs in cancer chemoresistance

The mechanisms through which circRNAs regulate the development of drug resistance in cancer are shown in **Tables 1 and 2**.

**Table 1:** CircRNAs affecting cell apoptosis in chemoresistance

Cancer	circRNAs	Role	Mechanism	Target/pathway	Drug	Ref
BC	circKDM4C	Down	miRNA sponge	miR-548p/PBLD	Doxorubicin	^39^
	hsa_circ_0006528	Up	miRNA sponge	miR-7-5p/Raf1	Doxorubicin	^40^
	CDR1as	Up	miRNA sponge	miR-7/CCNE1	5-FU	^41^
	circRNF111	Up	miRNA sponge	miR-140-5p/E2F3	Paclitaxel	^42^
	circABCB10	Up	miRNA sponge	let-7a-5p/DUSP7	Paclitaxel	^43^
Cervical cancer	hsa_circ_0023404	Up	miRNA sponge	miR-5047/BCL1/p62	Cisplatin	^44^
	circMTO1	Up	miRNA sponge	miR-6893/BCL1/p62	Cisplatin	^45^
CRC	hsa_circ_0007031	Up	miRNA sponge	miR-885-3p/AKT3/BCL2	5-FU	^46^
	hsa_circ_0000504	Up	miRNA sponge	miR-485-5p/AKT3/BCL2	5-FU	
	hsa_circ_0048234	Down	miRNA sponge	miR-671-5p/EGFR	5-FU	
	circPRKDC	Up	miRNA sponge	miR-375/FOXM1/Wnt/β-catenin	5-FU	^47^
	circCCDC66	Up	miRNA sponge	–	Oxaliplatin	^48^
GC	circPVT1	Up	miRNA sponge	miR-30a-5p/YAP1	Cisplatin	^49^
	circFN1	Up	miRNA sponge	miR-182-5p/caspase-3	Cisplatin	^50^
	hsa_circ_0000520	Down	-	PI3K/AKT	Herceptin	^51^
Glioma	circHIPK3	Up	miRNA sponge	miR-421/ZIC5	TMZ	^52^
				miR-524-5p/KIF2A/PI3K/AKT		^53^
HCC	hsa_circ_0003418	Down	miRNA sponge	miR-7/miR-383/Wnt/β-catenin	Cisplatin	^54^
	hsa_circ_101505	Down	miRNA sponge	miR-103/NOR1	Cisplatin	^55^
Lung cancer	hsa_circ_0085131	Up	miRNA sponge	miR-654-5p/ATG7	Cisplatin	^56^
	hsa_circ_0076305	Up	miRNA sponge	miR-296-5p/STAT3	Cisplatin	^57^
	hsa_circ_0007385	Up	miRNA sponge	miR-519d-3p/HMGB1	Cisplatin	^58^
	hsa_circ_0004350	Up	RBP sponge	EIF3a	Cisplatin	^59^
	hsa_circ_0092857	Up	RBP sponge	EIF3a	Cisplatin	
	hsa_circ_0096157	Up	–	p21/CCND1/CDK4/BCL2	Cisplatin	^60^
	CDR1as	Up	–	EGFR/PI3K	Cisplatin	^61^
					Pemetrexed	
	hsa_circ_0002483	Down	miRNA sponge	miR-182-5p/GRB2/FOXO1/FOXO3	Paclitaxel	^62^
	hsa_circ_0011292	Up	miRNA sponge	miR-379-5p/TRIM65	Paclitaxel	^63^
	hsa_circ_0004015	UP	miRNA sponge	miR-1183/PDPK1	Gefitinib	^64^
	hsa_circ_0003998	Up	miRNA sponge	miR-326	Docetaxel	^65^
	hsa_circ_0002130	Up	miRNA sponge	miR-498	Osimertinib	^66^
OS	hsa_circ_0001258	Down	miRNA sponge	miR-744-3p/GSTM2	Doxorubicin	^67^
					Cisplatin	
					Methotrexate	
	hsa_circ_0004674	Up	miRNA sponge	miR-1254/EGFR	Methotrexate	^68^
	hsa_circ_0000073	Up	miRNA sponge	miR-145-5p/miR-151-3p/NRAS	Methotrexate	^69^
	hsa_circ_001569	Up	–	Wnt/β-catenin	Cisplatin	^70^
Ovarian cancer	circCELSR1	Up	miRNA sponge	miR-1252/FOXR2	Paclitaxel	^71^
	circTNPO3	Up	miRNA sponge	miR-1299/NEK2	Paclitaxel	^72^
	CDR1as	Down	miRNA sponge	miR-1270/SCAI	Cisplatin	^73^
PCa	hsa_circ_0000735	Up	miRNA sponge	miR-7	Docetaxel	^74^
RCC	hsa_circ_0035483	Up	miRNA sponge	miR-335/CCNB1	Gemcitabine	^75^
Thyroid carcinoma	circEIF6	Up	miRNA sponge	miR-144-3p/TGF-α	Cisplatin	^76^
CML	hsa_circ_0009910	Up	miRNA sponge	miR-34a-5p/ULK1	Imatinib	^77^
	hsa_circ_0080145	Up	miRNA sponge	miR-326/PPFIA1	Imatinib	^78^
	circBA9.3	Up	–	c-ABL1/BCR-ABL1	TKIs	^79^
AML	circPAN3	Up	miRNA sponge	AMPK/mTOR	Doxorubicin	^80^
				miR-183-5p/XIAP		

**Table 2:** CircRNAs with other mechanisms in chemoresistance

Classification	Cancer	circRNAs	Role	Mechanism	Target/pathway	Drug	Ref
Drug excretion	CRC	hsa_circ_0005963	Up	miRNA sponge	miR-122/PKM2	Oxaliplatin	^84^
		hsa_circ_0007031	Up	miRNA sponge	miR-133b/ABCC5	5-FU	^88^
	GC	circMTHFD2	Up	miRNA sponge	miR-124/ABCC11	Pemetrexed	^89^
	Glioma	circCEP128	Up	miRNA sponge	miR-145-5p/ABCG2	TMZ	^90^
	Lung cancer	circPVT1	Up	miRNA sponge	miR-145-5p/ABCC1	Cisplatin	^86^
						Pemetrexed	
	OS	hsa_circ_0004674	Up	miRNA sponge	miR-490-3p/ABCC2	Doxorubicin	^68^
						Cisplatin	
		circPVT1	Up	–	ABCB1	Doxorubicin	^85^
						Cisplatin	
	OSCC	hsa_circ_0109291	Up	miRNA sponge	miR-188-3p/ABCB1	Cisplatin	^87^
	MM	hsa_circ_0007841	Up	–	ABCG2	Doxorubicin	^91^
DNA repair	BC	circMTO1	Down	RBP sponge	TRAF4/Eg5	Monastrol	^96^
	GC	circAKT3	Up	miRNA sponge	miR-198/PIK3R1	Cisplatin	^99^
		hsa_circ_0026359	Up	miRNA sponge	miR-1200/POLD4	Cisplatin	^100^
	Lung cancer	circPRMT5	Up	miRNA sponge	miR-4458/REV3L	Cisplatin	^102^
TME	BC	circUBE2D2	Up	miRNA sponge	miR-200a-3p	Tamoxifen	^105^
	GC	circPVT1	Up	miRNA sponge	miR-124-3p/ZEB1	Paclitaxel	^106^
	HCC	circMET	Up	miRNA sponge	miR-30-5p/snail/DPP4	Anti-PD-1	^107^
	Lung cancer	circFGFR1	Up	miRNA sponge	miR-381-3p/CXCR4	Anti-PD-1	^38^
		circCCDC66	Up	–	–	Cisplatin	^108^
CSC	Bladder cancer	circELP3	Up	–	–	Cisplatin	^114^
	CRC	hsa_circ_001680	Up	miRNA sponge	miR-340/BMI1	Irinotecan	^115^
	Lung cancer	circCPA4	Up	miRNA sponge	let-7/PDL1	Cisplatin	^116^
Endocrine	BC	hsa_circ_0025202	Down	miRNA sponge	miR-182-5p/FOXO3a	Tamoxifen	^122^
		circBMPR2	Down	miRNA sponge	miR-553/USP4	Tamoxifen	^123^
	PCa	hsa_circ_0004870	Down	RBP sponge	RBM39/AR-V7	Enzalutamide	^124^
		hsa_circ_0001427	Down	miRNA sponge	miR-181c-5p/ARv7	Enzalutamide	^125^
		circUCK2	Down	miRNA sponge	miR-767-5p/TET1	Enzalutamide	^126^

### CircRNAs affecting cell apoptosis in chemoresistance

Apoptosis is programmed cell death, which is regulated at the genetic level and removes damaged cells in an orderly and efficient manner^42^. The elimination of this death process is associated with unchecked cell proliferation, cancer development and progression, and cancer resistance to chemotherapies^43^. Consequently, dysregulation of molecules involved in apoptotic resistance often leads to treatment failure. According to extensive research, dozens of circRNAs downregulate apoptosis in cancer cells by regulating apoptosis-related genes, thereby leading to drug resistance. In the next paragraph, we briefly introduce the circRNAs that dysregulate apoptosis-related genes in cancers.

In breast cancer (BC), dysregulation of circKDM4C and hsa_circ_0006528 is associated with doxorubicin resistance. CircKDM4C, which is downregulated in BC, mitigates doxorubicin resistance by sponging miR-548p and targeting PBLD, a tumor suppressor in BC^44^. Additionally, hsa_circ_0006528, which is upregulated in doxorubicin-resistant cell lines, functions as an miRNA sponge *via* the miR-7-5p/Raf1 axis and contributes to doxorubicin resistance^45^. CDR1as is thought to promote 5-FU resistance in BC by competitively inhibiting miR-7 and consequently regulating CCNE1 expression^46^. CircRNF111^47^ and circABCB10^48^, both of which sponge miRNAs, induce paclitaxel resistance. In cervical cancer, hsa_circ_0023404 and circMTO1 both enhance the resistance to cisplatin and function as miRNA sponges that regulate beclin1 and p62^49,50^. In CRC, hsa_circ_0007031 and hsa_circ_0000504 promote 5-FU resistance by sponging miR-885-3p and miR-485-5p, and ultimately upregulate AKT3 and BCL2 expression. Moreover, hsa_circ_0048234 represses 5-FU resistance *via* the miR-671-5p/EGFR axis^51^. In addition, circPRKDC enhances resistance to 5-FU *via* the miR-375/FOXM1/Wnt/β-catenin pathway^52^. A recent study has revealed that circCCDC66 promotes oxaliplatin resistance in CRC by interacting with a set of miRNAs and consequently regulating the expression of multiple genes that facilitate cell apoptosis and survival. In addition, oxaliplatin induces circCCDC66 expression by phosphorylating DHX9, which is involved in multiple pathways associated with the DNA damage response^53^. In gastric cancer (GC), circPVT1^54^ and circFN1^55^ are significantly upregulated in paclitaxel-resistant GC tissues and cells, and these circRNAs have been predicted to regulate apoptosis *via* an miRNA sponge mechanism. Sun et al.^56^ have found that hsa_circ_0000520 enhances the sensitivity of herceptin in GC cells by targeting at PI3K/AKT. In glioma, circHIPK3 has been found to enhance cell proliferation and temozolomide (TMZ) resistance, not only by sponging miR-421/ZIC5^57^ but also *via* the miR-524-5p/KIF2A/PI3K/AKT axis^58^. In hepatocellular carcinoma (HCC), both hsa_circ_0003418^59^ and hsa_circ_101505^60^ enhance cell apoptosis and the sensitivity to cisplatin through an miRNA sponge mechanism. In lung cancer, 3 circRNAs leading to cisplatin resistance by sponging miRNAs have been identified: hsa_circ_0076305, hsa_circ_0007385, and hsa_circ_0085131^61–63^. Moreover, hsa_circ_0004350 and hsa_circ_0092857, both of which decrease cells’ sensitivity to cisplatin, can interact with RBPs and may have a synergistic effect with the parental EIF3a gene^64^. In addition, hsa_circ_0096157 enhances cisplatin resistance *via* regulating the p21/CCND1/CDK4/BCL2 apoptosis signaling pathway^65^, and CDR1as has been identified to contribute to both cisplatin and pemetrexed chemoresistance in lung adenocarcinoma *via* the EGFR/PI3K signaling pathway^66^. Other circRNAs functioning as miRNA sponges in lung cancer include hsa_circ_0002483^67^ and hsa_circ_0011292^68^ against paclitaxel, hsa_circ_0004015 against gefitinib^69^, hsa_circ_0003998 against docetaxel^70^, and hsa_circ_0002130 against osimertinib^71^. In osteosarcoma (OS), hsa_circ_0001258 has been found to decrease the resistance to doxorubicin, cisplatin, and methotrexate *via* miR-744-3p/GSTM2^72^. Furthermore, hsa_circ_0004674^73^ and hsa_circ_0000073^74^ repress cell apoptosis and resist methotrexate by sponging miRNAs, and hsa_circ_001569 represses cisplatin sensitivity *via* Wnt/β-catenin^75^. In ovarian cancer, the overexpression of circCELSR1^76^ and circTNPO3^77^ results in paclitaxel resistance by repressing cell apoptosis and regulating the cell cycle. In addition, CDR1as, in contrast to its role in BC and lung adenocarcinoma, is downregulated in cisplatin-resistant ovarian cancer tissues, and it decreases the resistance of cells to cisplatin *via* the miR-1270/SCAI axis^78^. In prostate cancer (PCa), Gao et al.^79^ have discovered that hsa_circ_0000735 overexpressed in PCa tissue, and downregulated of hsa_circ_0000735 boosts docetaxel sensitivity by promoting cell apoptosis. Hsa_circ_0035483 contributes to gemcitabine-induced autophagy and facilitates resistance of renal cell carcinoma (RCC) to gemcitabine *via* the miR-335/Cyclin B1 axis^80^; similarly, circEIF6 facilitates cisplatin resistance *via* miR-144-3p/TGF-α in thyroid carcinoma^81^. In chronic myeloid leukemia (CML), hsa_circ_0009910^82^ and hsa_circ_0080145^83^ repress cell apoptosis and sensitivity to imatinib by acting as miRNA sponges. CircBA9.3, which promotes proliferation and inhibits apoptosis in cancer cells, enhances resistance to tyrosine kinase inhibitors (TKIs) by increasing the expression of the c-ABL1 and BCR-ABL1 oncoproteins, which contribute to the immortality of leukemic cells^84^. CircPAN3, a circRNA highly expressed in doxorubicin-resistant acute myeloid leukemia (AML) cells, functions as an miRNA sponge that regulates autophagy and influences the expression of apoptosis-related proteins *via* the AMPK/mTOR signaling pathway, and ultimately contributes to doxorubicin resistance^85^. In general, circRNAs reverse drug sensitivity by regulating the expression of apoptosis-associated genes, thus repressing cancer cell death.

### CircRNAs promoting drug excretion in chemoresistance

The ABC transporter family comprises 48 genes subdivided into 7 subfamilies: ABCA through ABCG^86^. Most these genes encode large membrane proteins that function in the energy dependent transport of xenobiotics, metabolites, and signaling molecules across cell membranes, often against their concentration gradients^87^. Two ATP-binding sites in ABC transporters have been reported to participate in the formation of the ATP binding pocket, and their interactions are essential for coupling ATP hydrolysis to drug transport and ultimately cause chemoresistance^88^. According to recent studies, a group of circRNAs can target at ABC transporters and then promote drug excretion, thereby inducing drug resistance.

On the one hand, some circRNAs indirectly enhance the functionality of ABC transporters. Hsa_circ_0005963 has been found to promote oxaliplatin resistance *via* the miR-122/PKM2 axis^89^, thus enhancing glycolysis and ATP production and facilitating oxaliplatin excretion by ABC transporters in CRC. On the other hand, circRNAs can upregulate the ABC transporter family directly. Overexpression of hsa_circ_0004674 confers resistance to doxorubicin and cisplatin *via* the miR-490-3p/ABCC2 axis in OS^73^. CircPVT1 not only facilitates resistance to cisplatin and doxorubicin by enhancing the expression of ABCB1 in OS^90^, but also functions through miR-145-5p/ABCC1 in lung cancer, thus resulting in resistance to cisplatin and pemetrexed^91^. Hsa_circ_0109291, like circPVT1, targets ABCB1 by sponging miR-188-3p and consequently rendering cisplatin ineffective in oral squamous cell carcinoma (OSCC)^92^. He et al.^93^ have found that hsa_circ_0007031 represses 5-FU sensitivity *via* the miR-133b/ABCC5 axis in CRC. Xu et al.^94^ have demonstrated that circMTHFD2 enhances GC resistance to pemetrexed by sponging miR-124 and consequently increases the protein expression of ABCB11. CircCEP128 significantly promotes the expression of ABCG2 and resistance to TMZ by sponging miR-145-5p^95^ in glioma. In addition, in multiple myeloma (MM), overexpression of hsa_circ_0007841 leads to high levels of ABCG2 and consequently decreases doxorubicin’s efficacy^96^. These results suggest that circRNAs can directly or indirectly regulate the expression of ABC transporters and eventually induce drug resistance by promoting drug excretion.

### CircRNAs promoting DNA repair in chemoresistance

DNA repair is a biological event in which cells identify and correct DNA damage induced by chemotherapy and other incidents. The main mechanisms comprise base excision repair, nucleotide excision repair, mismatch repair, homologous recombination repair, nonhomologous end-joining, and interstrand crosslink repair, which can confer resistance to DNA-targeting chemotherapeutics^97^. Therefore, understanding the activity of different DNA repair pathways in individual tumors and the correlation between DNA repair function and drug response will be key in the selection of targeted drugs for patients. Because ncRNAs are a widespread concern, multiple circRNA-mediated DNA damage repair mechanisms leading to drug resistance have been discovered.

Eg5, a member of the kinesin family, whose members are crucial for maintaining separation of the half-spindles, is a microtubule-based motor protein required for the formation and maintenance of the bipolar spindle^98,99^. Monastrol, a reversible, cell-permeable small molecule, selectively inhibits Eg5 and consequently disrupts the structure of tumor cells’ chromosomes and DNA^100^. A recent study has identified a circular RNA, circMTO1, that is significantly downregulated in monastrol resistant cells and reverses monastrol resistance through RBP targeting at TRAF4/Eg5^101^.

Cisplatin, first synthesized by Michele Peyrone and approved by the US FDA for use in testicular and ovarian cancer in 1979^102^, binds DNA and forms adducts once inside tumor cells. The primary forms of DNA damage are N7-d (GpG) and N7-d (ApG) intrastrand DNA-platinum adducts, which cause substantial kinking of DNA^103^. In GC, both circAKT3 and hsa_circ_0026359 have been verified to induce cisplatin resistance. Huang et al.^104^ have identified that circAKT3 promotes DNA damage repair *via* miR-198/PIK3R1. Hsa_circ_0026359 sponges miR-1200 and upregulates POLD4^105^, and low expression of hsa_circ_0026359 has been reported to weaken DNA repair systems^106^. In addition, circPRMT5 overexpression in lung cancer enhances cisplatin resistance by upregulating REV3 L, which encodes the catalytic subunit of DNA polymerase ζ and is responsible for translesional replication^107^. In summary, circRNAs activate related proteins or pathways, thus maintaining chromosome or DNA stability, and promote DNA damage repair, thereby leading to chemotherapy failure.

### CircRNAs influencing the TME in chemoresistance

The TME, the cellular environment in which tumors exist, has been characterized as hypoxic, acidic, inflammatory, and immunosuppressive. Beyond tumor cells, the TME contains the extracellular matrix, surrounding blood vessels, and other non-malignant cells^38^. The TME is considered a “sanctuary of the devil,” in that immune cells in the TME secrete cytokines, inflammatory factors, and chemokines and drive the epithelial to mesenchymal transition (EMT) process in cancer cells *via* various pathways. In turn, cancer cells interact with immune cells and induce cellular plasticity and the release of immunosuppressive substances; consequently an immunosuppressive microenvironment that promotes immune escape and drug resistance is created^108^.

Several studies have shown that circRNAs alter the TME by regulating EMT and immune escape, and ultimately result in drug resistance. Hu et al.^109^ have reported that circUBE2D2 accelerates the progression of EMT and enhances resistance to tamoxifen in BC. CircPVT1, which is expressed at high levels in paclitaxel-resistant GC cells, sponges miR-124-3p and upregulates ZEB1, a crucial transcriptional inhibitor of E-cadherin that expedites EMT^110^. CircMET overexpression promotes an HCC immunosuppressive tumor microenvironment by inducing EMT through the miR-30-5p/Snail/DPP4/CXCL10 axis and leads to anti-PD1 therapy resistance^111^. In lung cancer, circFGFR1 has a critical role in immune evasion and induces resistance to anti-PD1 based therapy *via* the miR-381-3p/CXCR4 axis^112^. In addition, circCCDC66 promotes EMT and represses cisplatin sensitivity in lung adenocarcinoma^113^. Through this mechanism, circRNAs mainly alter the TME by regulating EMT, thus allowing cancer cells to escape drug and immune injury.

### CircRNAs regulating CSCs in chemoresistance

CSCs are a group of self-renewing cells that have high tumorigenic capacity and play crucial roles in chemoresistance, accelerating tumor regrowth after therapy^114^. CSCs share many characteristics with regenerative stem cells, such as self-renewal, multipotency, and the reversibility of their quiescent state^115,116^. After chemotherapy, residual bodies enriched in CSCs remain, which can promote tumor recurrence, re-grow metastatic tumors, and enhance resistance to drugs^117,118^.

To date, 3 circRNAs have been reported to influence CSC-like phenotypes. CircELP3, which is elevated in hypoxic environments, enhances cisplatin resistance in bladder cancer by displaying a high self-renewal capacity, as evidenced by high levels of sphere formation and stem cell marker expression^119^. Similarly, both hsa_circ_001680, resisting irinotecan in CRC^120^, and circCAP4, resisting cisplatin in lung cancer^121^, strengthen stem cell-like traits, self-renewal and tumor-initiating capacity *via* increasing stemness associated signatures and sphere formation. In conclusion, circRNAs increase cancer cells’ stemness, thus protecting tumors against chemotherapeutics.

### CircRNAs mediating endocrine therapy in chemoresistance

Endocrine therapy plays a unique role in the treatment of cancer, particularly in BC and PCa^122^. The estrogen receptor, which is expressed in approximately 70% of all BC, is considered the main factor inducing BC^123^. Tamoxifen, an anti-estrogen that decreases estrogen-induced effects by blocking the estrogen receptor in breast tissue, remains a cornerstone treatment that has significantly improved clinical outcomes^124^. Simultaneously, as an effective therapeutic target in PCa, the androgen receptor, is expressed in nearly all PCa and results in an abnormal gene expression profile, including cell cycle regulators, transcription factors, and genes important for cell survival, lipogenesis, and secretion^125^. Antiandrogenic drugs such as enzalutamide inhibit the progression of PCa by blocking androgens^126^. However, the development of drug resistance is inevitable after the first chemotherapy, and some circRNAs have been reported to participate in the progression of endocrine-related resistance.

In BC, 2 circRNAs that are downregulated in BC cells have been found to act synergistically with tamoxifen. Sang et al.^127^ have demonstrated that overexpressed hsa_circ_0025202 sponges miR-182-5p and upregulates FOXO3a, an inhibitor of tamoxifen resistance in BC. CircBMPR2 acts as an miR-553 sponge, thereby preventing it from repressing the tumor suppressor USP4, and subsequently mitigates tamoxifen resistance^128^. To date, downregulation of 3 circRNA in PCa cells has been reported to lead to increased resistance to enzalutamide. Low hsa_circ_0004870 expression results in the upregulation of its parental gene RBM39 and the downstream target gene AR-V7, whose expression is positively correlated with enzalutamide resistance^129^. Another circRNA that targets AR-V7 is hsa_circ_0001427. Interestingly, hsa_circ_0001427 is positively correlated with miR-181c-5p, which directly binds and degrades the ARv7 3′ untranslated region and may stabilize miR-181c-5p, thus ultimately decreasing cells’ resistance to enzalutamide^130^. Finally, CircUCK2, whose expression is downregulated in enzalutamide-resistant cells, may function as an miRNA sponge through the miR-767-5p/TET1 axis^131^. These results suggest that circRNAs regulate the effects of endocrine drugs by changing relative receptor levels, thereby potentially providing new directions in hormone-dependent cancer therapy.

## Discussion

Because of the vast amount of research on cancer, a variety of targeted chemotherapy drugs have been widely used in clinical settings and have achieved good efficacy. The most common mechanism of chemotherapy is inducing cancer cell death by damaging DNA to inhibit tumor growth. In contrast, the mechanisms of tumor drug resistance include promoting drug excretion and DNA repair, inhibiting apoptosis, and creating a TME resistant to the damage caused by chemotherapy drugs.

According to the literature, the multi-drug resistance of circRNAs may manifest different roles in different cancers. For example, CDR1as promotes resistance to cisplatin, 5-FU, and pemetrexed in BC and lung cancer, but it enhances the sensitivity of ovarian cancer to cisplatin. Although CDR1as appears to play a crucial role in the chemoresistance of cancers, whether an opposite effect might occur in different cancers, owing to the heterogeneity and origin of tumors, remains unknown. Moreover, reports on circRNA-related chemoresistance have largely focused on their mechanisms as miRNA sponges, which function *via* interactions between circRNA and miRNAs that regulate downstream genes and ultimately influence drug effects. In contrast, only few studies have reported the mechanisms of circRNAs as they relate to RBPs. Although circRNAs are known to regulate transcription and translation, these 2 mechanisms have not been reported in terms of circRNA-related drug resistance. Instead, dozens of studies have shown that ncRNAs influence chemoresistance by regulating transcription and translation; for example, miR-5787 affects the translation of mitochondrial cytochrome c oxidase subunit 3 (MT-CO3), thus regulating cisplatin resistance^132^, and miR-182-5p, regulates GLI2, a transcriptional regulator of the hedgehog signaling pathway^133^. Therefore, a knowledge gap regarding the mechanisms of circRNA-related drug resistance remains to be further explored.

Currently, RNA-seq, owing to its high throughput, accuracy, and sensitivity, is the gold standard for screening circRNAs associated with drug resistance. Most studies use RNA-seq to screen circRNAs with differential expression patterns between resistant and nonresistant cell lines, to explore mechanisms of chemoresistance. Interestingly, some studies have shown not only that circRNAs influence multi-drug resistance but also that drug feedback regulation on circRNAs exists. For example, circCCDC66 is a circRNA that is induced by treatment with oxaliplatin and subsequently promotes resistance to oxaliplatin-induced apoptosis. Future studies may reveal new mechanisms for the occurrence and enhancement of drug resistance. Blocking these mechanisms could substantially support the elimination of chemoresistance. This research direction is especially applicable to the clinical study of drug resistance and may provide new directions for the exploration of the mechanisms of circRNA-related chemoresistance.

With the recent development of liquid biopsy, examination of secretions, and other clinical techniques, circRNAs have broad application prospects as early screening indicators of drug sensitivity. For example, as mentioned above, hsa_circ_0025202 has an anti-oncogenic role in HR-positive breast cancer, and it may be exploited as a novel marker for tamoxifen-resistant breast cancer. Meanwhile, with research advances, many clinical trials are underway that are targeting ncRNAs, including a mimic of the tumor suppressor miRNA miR-34, which has reached phase I clinical trials^134^. Because circRNAs are emerging research targets, relevant clinical studies remain lacking, although many reports have already shown that circRNAs are associated with cancer progression and drug resistance, possibly because of the high toxicity and low specificity of the inhibitors, which are difficult to put into clinical use. In general, how to accurately target circRNAs to tumor cells and minimize the damage to healthy cells is an urgent problem that must be solved.

## Conclusions

In summary, circRNAs have been shown to be involved in tumor progression and to be closely associated with drug resistance. We summarized the mechanisms through which circRNAs interact with RBPs and miRNAs, and consequently influence drug delivery, reverse drug-induced apoptosis, and ultimately lead to drug resistance. However, many unknown circRNAs and mechanisms of drug resistance remain to be discovered and elucidated. Therefore, in-depth exploration in this field may provide a broader space for further research and treatment of chemoresistance mechanisms in tumors. circRNAs may have extensive roles in the treatment of tumors in the future.
